# Environmental Pollution Liability Insurance of Health Risk and Corporate Environmental Performance: Evidence From China

**DOI:** 10.3389/fpubh.2022.897386

**Published:** 2022-06-27

**Authors:** Wenqing Wu, Pianpian Zhang, Dongyang Zhu, Xin Jiang, Mihajlo Jakovljevic

**Affiliations:** ^1^College of Management and Economics, Tianjin University, Tianjin, China; ^2^School of International Education, Tianjin University, Tianjin, China; ^3^Institute of Advanced Manufacturing Technologies, Peter the Great St. Petersburg Polytechnic University, St. Petersburg, Russia; ^4^Institute of Comparative Economic Studies, Hosei University Chiyoda, Chiyoda, Japan; ^5^Department of Global Health Economics and Policy, University of Kragujevac, Kragujevac, Serbia

**Keywords:** environmental pollution liability insurance, health risk, environmental performance, public visibility, ownership structure

## Abstract

Environmental pollution liability insurance (EPLI) is a type of insurance purchased by an enterprise to compensate the loss of the victims in the event of an environmental pollution incident. Although EPLI can realize the post-treatment of environmental pollution to a certain extent, there is still less understanding of whether EPLI can improve the environmental performance of enterprises. This study takes A-share listed companies in heavily polluting industries as the research object, determines the treatment group samples according to the Insurance coverage list published by the Ministry of Environmental Protection in 2014 and 2015, and then constructs the empirical test model. In order to ensure that there is no sample selection bias, the PSM method is used to preprocess the samples in this study to ensure the robustness of the conclusions. The empirical tests show that EPLI can significantly improve corporate environmental performance. Further analysis showed that higher public visibility is conducive to the positive environmental effects of EPLI. Compared with state-owned enterprises, non-state-owned enterprises have more significant implementation effects after introducing EPLI. On further examination, the result indicates that environmental pollution liability insurance can improve environmental performance by alleviating corporate financing constraints. The findings of this paper enrich the theory of the economic impact of environmental pollution liability insurance, which has some meaningful theoretical guidance for enterprises and policy makers.

## Introduction

The massive urbanization in mainland China has raised issues related to industrial pollution affecting population health (1, 2). This is increasingly becoming one of the top priorities in governing authority's agendas (3, 4). It is clearly documented with a bold rise in Chinese domestic health and pharmaceutical spending in medium-term forecasted projections up to 2025 (5) and 2030 (6). The responsibility frontier in policy makers' mindset is now shifting from the public sector toward the private-owned manufacturing industry (7). The significance of corporate environmental performance in improving corporate value and corporate image has become increasingly important with the increasing concern of lawmakers and the public on environmental issues. Scholars began to pay more attention to what factors affect the environmental performance of enterprises. The existing literature mainly includes three aspects.First is the influence of internal factors of a company on its environmental performance, such as governance structure and CEO characteristics. For instance, Walls et al. (8) studied the influence of governance structure, including ownership, board size and managerial incentives, on corporate environmental performance. Elmagrhi et al. (9) found that with the increase in the proportion and age of female directors, a company's environmental performance would also increase. Slater and Dixon-Fowler (10) found that CEOs with MBA degrees bring a higher level of environmental performance to their companies. Second is the impact of external pressure on the company's environmental performance, such as regulation and social license. For instance, Kagan et al. (11) studied to what extent and how external factors such as regulation, market, and social pressure affect the environmental performance of corporates; Graafland and Smid (12) used SMEs as a research sample to explore how social licensing affects environmental performance. Third, the impact of policy tools on environmental performance, such as environmental management systems (EMS) and ISO 14001. For instance, Zobel (13) found that some certifications, such as EMS and ISO14001 certification, can effectively improve the environmental performance of enterprises. Environmental Pollution Liability Insurance (EPLI) not only has the function of post-compensation as a kind of insurance, but also shows its attribute as a system in the actual implementation. However, the existing research focuses more on the insurance nature of EPLI and ignores its institutional nature. There is a question worth discussing, that is, whether EPLI can effectively play the role of institutional norms to improve enterprises' environmental performance grade, and literature research on this aspect is still lacking.

Environmental pollution events often bring huge losses, both economically and environmentally. In recent years, the scope of responsibility of the principal responsible for pollution incidents has been expanding from international practice, which means that once the environmental pollution happens to the enterprise, the enterprise often has to face the huge capital repayment pressure. EPLI is a type of insurance used to compensate for injuries and deaths caused by pollution and related restoration and clean-up costs toward the third party. Therefore, EPLI can play a role in dispersing enterprise environmental risks. Scholars have conducted research on the effects of EPLI from different perspectives. On the one hand, some scholars compared the effects of EPLI in different implementation modes. Feng et al. (14) used a case study method to compare the effects of implementing compulsory and voluntary environmental pollution liability insurance, corresponding to Wuxi and Chongqing (China). The results showed that the local government could establish a relatively mature pollution insurance system more quickly with the implementation of compulsory insurance. On the other hand, some scholars have studied the role of EPLI. Staccione et al. (15) conducted interviews with experts and waste treatment and disposal plants (WTPs) operators to investigate their attitudes and perceived efficiency toward environmental insurance. The results showed that environmental insurance is a good financial tool for managing environmental risks. Yang et al. (16) took the enterprises in chemical industrial parks as an example to evaluate the implementation effect of EPLI to improve environmental risk management and made policy recommendations. In general, previous studies on the implementation effects of EPLI have focused less on enterprises and more on the insurance nature of EPLI. From the perspective of stakeholder theory, corporates should not merely focus on the interest of their shareholders, but also have to do their best to meet the expectation of other stakeholders. In the EPLI model, stakeholder relationships are formed between multiple roles, including governments, consulting teams, insurance companies, third party services and companies (17). Consulting teams will be responsible for designing the EPLI's operational mechanism; Governments will provide financial support and supervision for the operation of insurance; Insurance companies will collect premiums and make compensations when environmental pollution accidents occur; Third parties will provide on-site inspection and other environmental services. Under this system design, the common need of these external stakeholders for the company is to reduce the occurrence and loss of pollution incidents. Therefore, we believe that coverage of EPLI will help companies improve their environmental performance levels by increasing stakeholder pressure on companies. Previous literature did not examine the implementation effect of EPLI as a system. Given this research gap, we designed an empirical test in this paper to explore whether EPLI will have an impact on the environmental performance of companies.

Public visibility refers to the degree to which a company receives attention from the public (18). According to the stakeholder-agency theory, the problem of information asymmetry is widespread between management and stakeholders. This is because management can use their facilities to whitewash or selectively disclose internal information, which will increase costs for stakeholders to distinguish whether management decisions are in their favor. During the operation of the EPLI system, the acquisition of environmental information by stakeholders is an important link to ensure the implementation of environmental pressure on enterprises. Higher public visibility can assist stakeholders in determining whether a company's behavior is in line with their expectations (19). Combined with the above, the probability of corporate managers acting in the interests of their stakeholders' increases as their visibility increases. Therefore, we believe that a moderating role of public visibility is reflected in the moderating relationship between EPLI and corporate environmental performance.

Another influence of corporate environmental performance is corporate governance structure, such as ownership structure. The environmental performance of state-owned firms tends to be higher than that of non-state-owned enterprises because the goal of state-owned firms is to maximize economic welfare (20). EPLI improves corporate environmental performance by introducing stakeholder groups to exert environmental pressure on companies. According to resource dependence theory, the pressure exerted by stakeholders on a firm depends on the importance of the resources they control to the firm. State-owned enterprises can obtain external resources more easily by virtue of their political connections. Malatesta and Dewenter (21) found it easier for politically connected companies to obtain debt financing. Therefore, we believe that non-state-owned firms are more sensitive to environmental pressures from EPLI and have more incentive to improve environmental performance than state-owned enterprises.

As the largest developing country, the Chinese government has paid particular attention to environmental issues in recent years (22). The government has also supported EPLI. China's EPLI was officially introduced in 2007. The Guidance on Environmental Protection and the Insurance Regulation Commission clarified the objective orientation, development principle, division of responsibilities and specific work content arrangement of EPLI in China. To address the above research gaps, we examine the institutional effects of EPLI and focus on whether EPLI can positively affect firms' environmental performance by combining stakeholder theory, agency theory, and resource dependence theory. In addition, we further investigate the moderating effect of public visibility and ownership structure from the perspective of stakeholder pressure. Moreover, this paper takes 2014–2015 insured companies published by the Ministry of Environmental Protection as data collection objects and conducts an empirical study on listed companies in heavy pollution industries in China to test our theoretical hypothesis. Our research objectives include (a) identifying the impact and mechanism of EPLI on corporate environmental performance, (b) examining the moderating effect of firm visibility on the relationship between EPLI and corporate environmental performance, and (c) examining the moderating effect of ownership structure on the relationship between EPLI and corporate environmental performance.

The contributions of this study are: First, this paper enriches the literature on the microeconomic effects of EPLI and uses empirical methods to explore the impact of EPLI on corporate environmental performance. Second, this study focuses on the institutional effects of EPLI, which enriches the theoretical research on the effects of EPLI's implementation. Third, this paper uses stakeholder theory as the main theoretical support, combined with agency theory and resource dependence theory, to construct a theoretical framework to explain the effect of EPLI on corporate environmental performance, enriching the connotation of existing theories.

The remainder part of this paper is structured as follows. The following section will introduce the relevant research on EPLI and environmental performance, theoretical background and hypothesis derivation. In the following chapters, we will report the design and the findings of our empirical research. The last part will summarize the whole study and put forward optimization suggestions.

## Theoretical Background and Hypothesis

It can be found from the dimension of stakeholder theory that since the needs of stakeholders are different and sometimes contradictory, managers will respond to the needs of stakeholders according to certain priorities, which are determined by stakeholder salience. Stakeholder salience can be described as the degree of pressure imposed by stakeholders on management, which is the function of power, legitimacy and urgency (23). The central stakeholders in China's EPLI system are insurance companies and the government (17). On the one hand, a contractual relationship is formed between enterprises and the insurance companies, and the circumstances that trigger a change in the interests of both parties are pollution accidents because they will lead directly to insurance claims. On the other hand, the roles of government for enterprises are administrative support and supervision. With the introduction of the enactment in China linking the performance evaluation of local officials to environmental issues in 2006, environmental accidents will directly affect the promotion benefits of officials. Therefore, both insurance companies and the government have a power-basis, legitimate and urgent needs to improve corporate environmental performance. Further, the insurance companies and the government will be classified as definitive stakeholders because they meet all three attributes according to the stakeholder salience theory, whose demands will put more pressure on managers than other stakeholders' demands (23). Studies have found that high pressure from stakeholders can promote the growth of corporate environmental performance (24).

Furthermore, according to stakeholder-agency theory, adequate disclosure of internal information will increase the pressure on managers to act in line with stakeholders' interests. EPLI can alleviate the degree of information asymmetry by introducing external supervision. On the one hand, the governance structure of China's EPLI system is generally dominated by the government (25), and the government will supervise and evaluate the effect of the implementation of the EPLI. On the other hand, third-party service agencies will also provide on-site inspection and other services to supervise the effectiveness of the system (17). In the process of supervising the enterprise, stakeholders in the EPLI system will make the information on the firm's environmental performance more widely spread among them (14), which will increase the environmental pressure on the firm.

In summary, EPLI can alleviate the problem of information asymmetry between major stakeholders in the EPLI system and enterprises to a certain extent and thus increase the pressure on managers to improve environmental performance. Based on the above discussion, we propose our first hypothesis:

**Hypothesis 1**. EPLI has a positive impact on corporate environmental performance.

According to stakeholder-agency theory, even though enterprises are faced with pressure from stakeholders, the management still tends to engage in opportunistic behaviors that are inconsistent with the expectations of stakeholders. However, more visible companies will face more burdensome external constraints and higher public demands for corporate citizenship, which will set a higher threshold for managers' opportunistic behavior (18).

On the one hand, widespread public attention can help stakeholders in the EPLI system determine whether a company's activities meet green standards (19). Companies with high public visibility will attract more public attention, which means that when companies purchase EPLI, there will be more third-party organizations such as media and securities analysts to report and evaluate this event (26). In other words, the attention of public institutions has broadened the channels for stakeholders to access information related to the company's purchase of EPLI. Therefore, public visibility will help stakeholders judge whether the actions of managers are in their interests and thus further increase the environmental pressure of stakeholders on the company.

On the other hand, with the increase in visibility, the company is faced with pressure from the public, a potential stakeholder, which urges the company to participate more in social responsibility activities (27). Flammer (28) also found that external green pressure from public concern will lead to the formation of green social responsibility norms. This means that stakeholders in the EPLI system will put more environmental pressure on the company when the company's public visibility is higher, thus making the contribution of EPLI to the company's environmental performance stronger. In summary, greater public visibility will curb the opportunistic behavior of managers, thus increasing the pressure of stakeholders in the EPLI system on companies to improve their environmental performance. Based on the above discussion, we propose our second hypothesis:

**Hypothesis 2**. Public visibility plays a positive role in moderating the relationship between EPLI and corporate environmental performance.

Existing research provides evidence for the relationship between ownership structure and environmental performance (29). State-owned enterprises (SOEs) have more political ties than non-SOEs, and the influence of such ties is stronger than in other countries due to the particularities of China's market economy development (30). For non-SOEs, environmental pressure exerted by stakeholders has a more significant impact on its environmental performance. On the one hand, Chinese SOEs have better access to external debt financing and government subsidies (31). According to resource dependence theory, when the resources held by stakeholders cannot pose a threat to the company, the power of stakeholders on managers will also be weakened. Therefore, the stakeholder pressure brought by EPLI will not significantly affect the company's willingness to improve its environmental performance in the context of relatively loose external restrictions of SOEs.

On the other hand, SOEs face more political intervention to engage in more socially beneficial activities (32, 33). This makes SOEs pay more attention to avoiding adverse social impacts, which means that as SOEs face more significant political pressure to improve their environmental performance, the positive role of EPLI will become less significant. Therefore, compared with non-SOEs, EPLI has no significant effect on the environmental performance improvement of SOEs. Based on the above discussion, we propose our third hypothesis:

**Hypothesis 3**. Compared with SOEs, EPLI has a more significant positive effect on corporate environmental performance in non-SOEs.

## Materials and Methods

This study selected heavily polluting industry companies in the A-share market listed on the Shanghai and Shenzhen exchanges as our sample. The reason for selecting companies in heavily polluting industries as samples is that most companies in the insurance coverage list are from heavily polluting industries. Focusing on heavily polluting industries can eliminate the problem of sample selection bias to a certain extent.

According to 2003, 2008, and 2012 classification standards of heavily polluting industries announced by the Ministry of Environmental Protection and the listed company classification guidelines announced by the China Securities Regulatory Commission in 2012, we selected a total of 44 industries, including the metal products industry, pharmaceutical manufacturing industry, chemical raw materials and chemical products manufacturing industry as our target industries. We screened the listed companies in these industries according to the following criteria: 1. Exclude listed companies that regulators give special treatment (ST) because of questions about the authenticity of their financial data. 2. Eliminate the missing company samples of key variables. We ended up with a total of 912 company-year observations, of which EPLI covered 116 samples. The EPLI coverage data is manually collected according to the Insurance coverage list announced by the Ministry of Environmental Protection in 2014 and 2015 and the iFind database. The company's financial data comes from the China Stock Market and Accounting Research (CSMAR) financial database. The environmental performance data comes from Rankins CSR rating database. Statistics software is Stata 15.0.

### Variables

#### Dependent Variable

##### Corporate Environmental Performance (CEP)

There are two options for measuring environmental performance in the existing literature: The first one is taking the company's pollutant emission level as the measurement standard. For example, Quying (34) adopted the ratio of expense on pollutant emission to operating revenue as a proxy variable for environmental performance. Ren et al. (35) measured the environmental performance based on changes in emissions of waste gas, wastewater and solid waste. Similarly, some other literature has also adopted quantitative indicators to measure CEP (36, 37). The advantage of using quantitative data to measure environmental performance is that the data is more reliable, but the limitation is that it only focuses on the dimension of corporate emissions and ignores the importance of environmental strategy. The second one is using qualitative indicators such as scoring to measure CEP. Klassen and McLaughlin (38) applied positive environmental events to represent good environmental performance and negative events to represent poor environmental performance. Griffin et al. (39) constructed environmental performance indicators based on corporate environmental strength and concern levels in data sets such as MSCI, ESG, KLD, and STATS. In the study of Wang et al. (40), the “green watch” project supported by the World Bank was introduced, which applies to China's corporate environmental performance rating. The rating system covers emission standards, whether it has passed ISO14000 or not, and divides CEP into five grades. This indicator has also been applied in the empirical study of Duanmu et al. (41). However, the implementation degree of the “green watch” project varies in different provinces, so it is not a suitable choice when testing with A-share listed companies as the sample.

In this paper, we chose the RKS ratings to measure the company's environmental performance from the RKS dataset because we believe it can reflect a company's environmental performance more comprehensively (9). RKS is currently the only third-party rating indicator in China. It is based on the KLD framework and GRI 3.0 global reporting standards, and uses 70 indicators to analyze and score the content of various social responsibility reports issued by listed companies in China. The ratings range from 0 to 100 and are evaluated from the dimensions of social responsibility strategy and innovation, disclosure content, and technical sufficiency.

#### Independent Variable

##### Environmental Pollution Liability Insurance (EPLI)

Since the firm's decision to purchase Environmental Pollution Liability Insurance for the Group and its subsidiaries does not require the consent of the board of directors, it is not feasible to obtain insurance coverage data totally from public information disclosed by listed companies. We finally selected the insurance coverage list announced by the Ministry of Environmental Protection (only published in 2014 and 2015) as the primary data source and supplemented by the public information disclosure of listed companies in the iFind database. We adopted a dummy variable (Ins) to measure EPLI, which equals 1 if the company is insured that year; otherwise, 0.

#### Public Visibility

We measured public visibility (Vis) by the percentage of revenue a company spent on advertising (42). The company's investment in advertising is conducive for consumers and investors to understand the company's brand and products better so that the company will be able to attract wider public attention (43). Therefore, we consider the size of advertising spending to be an intuitive measure of a company's public visibility. Specifically, we use the ratio of advertising expenses (e.g., advertising, exhibition, publicity, etc.) included in sales expense to sales revenue as a proxy variable for public visibility.

#### Control Variables

Previous studies on environmental performance examined the role of some company characteristics. To avoid interference of other factors in our observed relationship between EPLI and CEP, we controlled for the following factors in our model. Specifically, we selected firm size, leverage, return on asset (ROA), management expense ratio, firm age and property nature as our control variables. Each variable is explained as follows:

Firm size (*Size*): According to stakeholder theory, larger companies often face greater stakeholder pressure. They also control more resources to ensure they can engage in activities that improve environmental performance (44). Therefore, we assume that firm size will be related to environmental performance. Referring to the relevant empirical literature, we used the natural logarithm of the total assets of the company as the proxy variable of company size (45).

Leverage (*Lev*): The asset-liability ratio reflects a company's capital structure and financial condition. The existing empirical studies show that the leverage ratio reflects the pressure of the company to bear from the creditors and thus positively affects the company's environmental performance. However, Cormier and Magnan (46) found that the leverage negatively affected corporate environmental information disclosure. Considering the above empirical results, we included leverage as a control variable.

Return on asset (*ROA*): ROA measures a company's financial performance. The company's profitability will affect the resources that the company can invest to improve the environmental performance and thus have an influence on the environmental performance. We used the net profit ratio to weighted average total assets to calculate ROA in this article.

Management expense ratio (*GA*): The ratio of administrative expenses to operating income. The management expense ratio is also an indicator of the company's financial performance. We take this index as the control variable in this paper.

Firm age (*Age*): Referring to the empirical study of Cole et al. (47), we chose company age as the control variable. We assume that younger companies are more environmentally conscious and more willing to use cleaning equipment. We define this variable as the natural logarithm of the number of years since the company was founded.

Ownership nature (*SOE*): China's state-owned enterprises often face stronger institutional pressure to improve their environmental performance (9, 48). The empirical study of Earnhart and Lizal (49) also showed that the increase in state-owned ownership has a positive impact on environmental performance. Specifically, we assigned a value of 1 to the state-owned enterprises and a value of 0 to the non-state-owned enterprises in our sample.

Corporate transparency (*EID*): Corporate transparency can be defined as the extent to which a corporate discloses important management and operation information to the outside world. Greater firm transparency means that companies are devoting more resources to addressing information asymmetry with their stakeholders (18). We believe that corporate transparency reflects the extent to which companies take proactive steps to reduce information asymmetry, while EPLI reduces information asymmetry through the active behaviors of stakeholders. Therefore, we apply corporate transparency as the control variable. We referred to Xia et al. (50) and adopted the level of Environmental Information Disclosure (EID) to measure corporate transparency. EID is the method of project scoring. The specific scoring items are shown in Table 1. Each item is granted 3, 2, 1, or 0 points depending on the disclosure of financial information, specific non-monetary information, and general non-monetary information. The final score of EID is the sum of the scores of 10 items.

**Table 1:** Measurement items for EID.

**Item**	**Content**
I_1_	Enterprise environmental protection investment and environmental technology development
I_2_	Government grants, financial subsidies and tax breaks related to environmental protection
I_3_	Discharge of pollutants from enterprises and emission reduction
I_4_	ISO environmental system certification information
I_5_	Measures to improve the ecological environment
I_6_	The impact of government environmental policy on enterprises
I_7_	Loans for environmental protection
I_8_	Litigation, compensation, fines and awards related to environmental protection
I_9_	The concept and goal of enterprise environmental protection
I_10_	Other income and expenditure items related to the environment

#### Models

The basic hypothesis required for testing is that the EPLI has a positive effect on the CEP. The basic model applied is:


(1) $$\begin{array}{l} CEP_{i,t}\;\;=\;\;\beta_{0}\;+\;\beta_{1}\;Ins_{i,t}\;+\;\beta_{2}\;Control\;Variables{\;}_{i,t}\\ \;+\sum^{\text{​}}Year\;+\;\sum^{\text{​}}Industry\\ \;\begin{array}{c} +\;\sum^{\text{​}}Region+\varepsilon_{it} \end{array} \end{array}$$

Where *i* is for individual corporate and *t* for the year, *CEP* is the corporate's environmental performance; *Ins* is a dummy variable representing whether the company is insured for EPLI. If the company insured EPLI in the current year, the value of *Ins* is equal to 1; otherwise is equal to 0. *Control Variables* include *Size, Lev, ROA, GA, Age*, and *SOE*; β_*i*_ is the model regression coefficient; ε_*it*_ is the residual term. Furthermore, we added annual, regional, and industry dummy variables to the model to control for fixed effects.

In order to test the moderating effect on public visibility to the relation between EPLI and CEP, we adopted the following model for verification.


(2) $$\begin{array}{l} \begin{array}{c} \;CEP_{i,t}\;=\;\beta_{0}\;+\;\beta_{1}\;Ins_{i,t}\;+\;\beta_{2}Vis_{i,t}\;+\\ \beta_{3}Ins^{*}Vis\;+\;\beta_{4}Control\;variables\;+\;\sum^{\text{​}}\;Year\;+ \end{array}\\ \;\begin{array}{c} \sum^{\text{​}}Industry\;+\;\sum^{\text{​}}Region\;+\;\varepsilon_{it} \end{array} \end{array}$$

Model 2 adds public visibility variables (*Vis*) and the interaction term of EPLI and public visibility (Ins^*^Vis) based on Model 1. *Ins* is a categorizing variable, and *Vis* is a continuous variable. We can judge the moderating effect of public visibility when the company is insured EPLI (the value of *Ins* is equal to 1) by testing the significance of the interaction term coefficient. Regarding the moderating effect of the ownership structure, this paper tests it through group regression.

## Results

### Descriptive Analysis

Statistics for critical variables of the model are reported in Table 2, including the number of observations, mean, median, standard deviation, maximum and minimum. The mean value of *Ins* is 0.127, which means that 12.7% of the sample observations were insured against EPLI. It can be seen that the EPLI coverage rate of listed companies is generally low. The mean value of variable *CEP* is 40.664, and the median value is 38.256, indicating that the environmental performance of sample companies is generally higher than the average level. In addition, the standard deviation of the variable *CEP* is 10.94, which is significantly higher than other variables, showing the strong heterogeneity of the environmental performance of sample companies. The mean value of *SOE* is 0.593, indicating that state-owned enterprises in the sample account for the majority. The standard deviations of the variables that reflect a company's financial performance (*ROA, GA*) are 0.082 and 0.492, respectively. It indicates that the volatility of variable ROA is stronger than that of GA.

**Table 2:** Descriptive statistics of variables.

**Variable**	**Obs.**	**Mean**	**Median**	**Std. Dev**.	**Min.**	**Max.**
CEP	912	40.664	38.256	10.940	18.272	87.948
Ins	912	0.127	0	0.333	0	1
Size	912	22.980	22.889	1.758	12.746	28.509
Lev	912	0.488	0.497	0.207	0.009	1.037
ROA	912	0.040	0.034	0.059	−0.645	0.265
GA	912	0.086	0.071	0.082	0.002	1.178
Age	912	2.816	2.833	0.389	1.609	7.608
SOE	912	0.593	1	0.492	0	1
EID	912	4.162	3	4.054	0	20
Vis	479	0.752	0.008	4.999	−0.037	79.683

### Correlation Analysis

The correlation coefficient matrix reflecting the correlation between variables is reported in Table 3. We can see that the variables *CEP* and *Ins* show a positive correlation at the level of 0.01, which preliminarily confirms hypothesis 1, assuming that EPLI has a promoting role on CEP. The correlation coefficient between the variables *CEP* and *SOE* is significantly positive, which also reflects that the environmental performance of state-owned enterprises is better.

**Table 3:** Correlation coefficient matrix.

**Variables**	**CEP**	**Ins**	**Size**	**Lev**	**ROA**	**GA**	**Age**	**SOE**	**EID**	**Vis**
CEP	1									
Ins	0.144^***^	1								
Size	0.358^***^	0.123^***^	1							
Lev	0.117^***^	0.049	0.471^***^	1						
ROA	0.024	−0.014	−0.061^*^	−0.408^***^	1					
GA	−0.097^***^	−0.049	−0.273^***^	−0.375^***^	−0.023	1				
Age	−0.035	0.007	0.056^*^	0.125^***^	−0.067^**^	−0.095^***^	1			
SOE	0.181^***^	0.035	0.230^***^	0.259^***^	−0.213^***^	−0.181^***^	0.159^***^	1		
EID	0.072^**^	0.154^***^	0.116^***^	0.054^*^	−0.150^***^	−0.103^***^	−0.013	0.126^***^	1	
Vis	0.169^***^	0.152^***^	0.113^**^	0.078^*^	0.006	−0.017	0.185^***^	−0.077^*^	−0.005	1

### Regression Analysis Results

The regression results of Models 1 and 2 are reported in Table 4. The values in parentheses represent the t value of the coefficient of the variables. Hypothesis 1 proposed that the environmental performance of corporates will be significantly enhanced under the influence of EPLI. We regressed the corporate's environmental performance to the EPLI and control variables with robust standard errors clustered at the corporate level in Model 1. The results showed that EPLI is positively correlated with a corporate environmental performance at the significance level of 5%, suggesting that the company's environmental performance can be improved by insuring EPLI.

**Table 4:** Model regression results (1).

	**Model 1**	**Model 2**
**Variables**	**CEP**	**CEP**
EPLI	2.920^**^	0.863
	(1.423)	(1.430)
Lev	3.695	−3.448
	(3.183)	(5.219)
EID	−0.142	−0.024
	(0.109)	(0.176)
ROA	6.029	3.421
	(6.989)	(8.973)
SOE	2.500^**^	3.442^**^
	(1.038)	(1.415)
GA	−3.834	−5.800
	(5.671)	(9.814)
Age	−2.088^**^	−1.797
	(0.932)	(1.100)
Size	1.605^***^	2.405^***^
	(0.379)	(0.663)
Vis		−0.058
		(0.092)
Ins^*^Vis		0.560^***^
		(0.151)
Industry FE	Control	Control
Year FE	Control	Control
Region FE	Control	Control
Constant	13.260	−5.414
	(9.039)	(16.000)
Observations	912	479
R-squared	0.373	0.450
*F*	6.270	4.530

*Robust standard errors in parentheses*.

*** *p < 0.01,*

** *p < 0.05,*

* *p < 0.1*.

The regression results of model 2 show that the interaction term coefficients of Ins and Vis are positive and significant at the 1% level. As described in the previous model setting section, if the interaction term coefficient is significantly positive, we can reasonably assume that public visibility can expand the impact of EPLI on corporate environmental performance. In other words, the higher the public visibility of the company, the deeper the impact of EPLI on the company's environmental performance.

We divided the samples into groups of SOEs and groups of non-SOEs and conducted regression, respectively, to test the moderating effect of the company's ownership structure. The regression results are reported in Table 5. The *p*-value of the coefficient test of variable *Ins* of the non-state-owned enterprises' group is 0.051, while the *p*-value of the state-owned enterprise group is 0.147. This result indicates that EPLI has little effect on the improvement of the environmental performance of state-owned enterprises. In contrast, for non-state-owned enterprises, EPLI is an effective means to improve their environmental performance, which supports hypothesis 3.

**Table 5:** Model regression results (2).

	**Non-state-owned enterprises**	**State-owned enterprises**
**Variables**	**CEP**	**CEP**
EPLI	5.072^*^	2.408
	(2.579)	(1.654)
Lev	3.409	6.883
	(4.667)	(4.596)
Size	1.274^***^	1.717^***^
	(0.469)	(0.595)
ROA	16.970^*^	9.323
	(8.637)	(10.600)
GA	−2.511	8.434
	(6.822)	(13.450)
EID	0.040	−0.212
	(0.162)	(0.146)
Age	−2.700	−2.336^**^
	(2.020)	(1.016)
Constant	18.950^*^	14.410
	(11.020)	(14.330)
Industry FE	Control	Control
Year FE	Control	Control
Region FE	Control	Control
Observations	371	541
R-squared	0.441	0.403
*F*	3.500	4.520

*Robust standard errors in parentheses*.

*** *p < 0.01,*

** *p < 0.05,*

* *p < 0.1*.

### Robustness Checks

Due to the low coverage rate of EPLI (12.7%) in the samples we used, the empirical test with such samples may lead to the problem of sample self-selection. We adopted the propensity score matching (PSM) procedure to process the samples. The aim is to match a group of samples with the most similar propensity score for those who purchase EPLI. Specifically, we matched the samples based on three key variables: company size, ROA and the number of years of company listing (1:2 matching). The differences in critical variables between the control and treatment groups before and after matching are shown in Table 6. It can be seen that except for *EID*, other variables are not significant in the *t*-test after matching, indicating that the matching effect is good. We used the matched samples for the model test, and the results were reported in Table 7. The results were consistent with the conclusions of our empirical test before. Therefore, we believe that our conclusions in the empirical test section are robust.

**Table 6:** Sample balance test.

	**Unmatched**	**Mean**		***t*** **-test**		
**Variable**	**Matched**	**Treated**	**Control**	**% bias**	***t***	***p*****> |** ***t*** **|**
Lev	U	0.515	0.484	15.700	1.480	0.140
	M	0.517	0.536	−9.800	−0.720	0.469
Size	U	23.547	22.897	34.400	3.750	0.000
	M	23.640	23.643	−0.200	−0.020	0.987
ROA	U	0.038	0.040	−4.500	−0.420	0.678
	M	0.036	0.044	−13.000	−1.050	0.293
EID	U	5.802	3.923	46.200	4.720	0.000
	M	5.704	4.091	39.700	2.950	0.003
SOE	U	0.638	0.587	10.500	1.050	0.294
	M	0.643	0.591	10.700	0.810	0.418
GA	U	0.075	0.087	−17.500	−1.480	0.139
	M	0.075	0.073	2.500	0.240	0.809
Age	U	2.823	2.815	2.200	0.210	0.837
	M	2.829	2.862	−9.400	−0.560	0.577

**Table 7:** Robustness test (1).

	**Model 1**	**Model 2**
**Variables**	**CEP**	**CEP**
EPLI	3.132^*^	0.659
	(1.701)	(1.886)
Lev	1.854	11.720^*^
	(5.471)	(6.838)
EID	−0.027	0.482
	(0.213)	(0.314)
ROA	7.500	18.030
	(14.800)	(22.690)
SOE	3.719^**^	3.647
	(1.649)	(2.315)
GA	22.960^*^	7.667
	(12.780)	(12.310)
Age	−3.495^**^	−3.296^**^
	(1.356)	(1.478)
Size	2.575^***^	−0.406
	(0.768)	(1.092)
Vis		−0.023
		(0.129)
Ins^*^Vis		0.361^**^
		(0.149)
Constant	−8.510	56.920^**^
	(17.300)	(23.920)
Industry FE	Control	Control
Year FE	Control	Control
Region FE	Control	Control
Observations	297	143
R-squared	0.550	0.793
*F*	3.94	5.40

*Robust standard errors in parentheses*.

*** *p < 0.01,*

** *p < 0.05,*

* *p < 0.1*.

Considering that the possible inverse causality between environmental performance and EPLI may bring about the problem of endogeneity in the model, we construct the model using the explained variables 1 year in advance for regression. The reason for choosing the explained variable 1 year in advance is that our explanatory variable has only two data periods. The model used for the robustness test is shown as follows:


(3) $$\begin{array}{l} \begin{array}{c} CEP_{i,t\;+\;1}\;=\;\beta_{0}+\beta_{1}Ins_{i,t}\;+\;\beta_{2}Control\;Variables_{i,t}\;+ \end{array}\\ \begin{array}{c} {\;\sum }^{\text{​}}\;Year+\sum^{\text{​}}Industry\;+\;\sum^{\text{​}}Region\;+\;\varepsilon_{it} \end{array} \end{array}$$

(4) $$\begin{array}{l} \begin{array}{c} CEP_{i,t\;+\;1}\;=\;\beta_{0}\;+\;\beta_{1}Ins_{i,t}\;+\;\beta_{2}\;Vis{\;}_{i,t}\;+\;\beta_{3}Ins^{*}Vis\;+\\ \;\beta 4\;Control\;variables\;+\;\sum^{\text{​}}\;Year \end{array}\\ \begin{array}{c} \;+\;\sum^{\text{​}}Industry\;+\;\sum^{\text{​}}Region\;+\;\varepsilon_{it} \end{array} \end{array}$$

We controlled the models' fixed effects of industry, year and region, effectively avoiding the endogenous problem caused by missing variables. The test results of Models 3 and 4 are reported in Table 8. There is no material difference between our results and the above. Therefore, we believe that our conclusions obtained in the empirical test are robust.

**Table 8:** Robustness test (2).

	**Model 5**	**Model 6**
**Variables**	**CEP**	**CEP**
EPLI	2.490^*^	1.596
	(1.463)	(1.640)
Lev	4.189	−4.576
	(3.441)	(5.097)
EID	−0.154	−0.059
	(0.108)	(0.174)
ROA	7.482	5.635
	(7.397)	(10.700)
SOE	1.896^*^	2.439
	(1.093)	(1.510)
GA	−7.328	−7.150
	(6.077)	(11.430)
Age	−0.898	−0.267
	(1.058)	(1.032)
Size	1.691^***^	2.814^***^
	(0.446)	(0.638)
Vis		0.043
		(0.092)
Ins^*^Vis		0.367^***^
		(0.139)
Constant	12.540	−13.510
	(10.190)	(15.030)
Industry FE	Control	Control
Year FE	Control	Control
Region FE	Control	Control
Observations	875	457
R-squared	0.392	0.485
*F*	6.490	4.930

*Robust standard errors in parentheses*.

*** p < 0.01, ^**^p < 0.05,

* *p < 0.1*.

## Discussion

In view of the widespread concern about the green issue, the environmental responsibility of enterprises, especially the heavily polluting ones, is becoming increasingly important (51, 52). In practice, terrible performance on green social responsibility will hurt the corporate reputation and core competitiveness, thus undermining the value of a company (53, 54). In previous literature studies, EPLI has been studied more as a tool for environmental compensation. In fact, EPLI shows its institutional nature in the actual design and operation process, which means that EPLI is likely to play a further role in improving the environmental performance of enterprises; however, the research on this aspect is still lacking. The current study aimed to fill in the gaps in the existing literature on the effect, mechanism and influencing factors of EPLI on corporate environmental performance (55).

Using panel data from listed companies in China's heavily polluting industries from 2014 to 2015, we examined whether and how EPLI affects companies' environmental performance. Our empirical results showed the following findings. First, our results indicated that EPLI has a positive impact on corporate environmental performance. This discovery extended the research conclusions of Yang et al. (17) and provided empirical evidence of the effectiveness of EPLI operation in China. From the perspective of stakeholder theory, the formation of the new stakeholder relationship will lead to changes in the pressure exerted by stakeholders on the company, thus changing the company's environment, practices and strategic choices. After the company purchases EPLI, it forms a new interest relationship among enterprises, government and insurance companies (17). Through the risk transfer mechanism of EPLI products, the losses caused by pollution events will be directly related to the stakeholders in this system. Therefore, EPLI will increase the urgent pressure of stakeholders' environmental demands on company managers, thus prompting managers to adopt green measures to improve the company's environmental performance.

Second, we found that public visibility positively moderates the relationship between EPLI and corporate environmental performance. This finding revealed that EPLI is more effective in improving the environmental performance of companies with higher public visibility, which is in line with the findings of Wu et al. (56). As Dou et al. (18) indicated, the public concern has raised higher requirements for the legitimacy and citizenship of enterprises. Therefore, in a more visible corporate environment, managers' opportunistic behavior will be severely constrained, leading them to act in accordance with stakeholder expectations. Moreover, the widespread public attention will broaden the channels for stakeholders to obtain relevant information about the company and help stakeholders judge whether the company's actions truly serve their interests, which also negatively affects managers' opportunistic behavior.

Third, we found that EPLI has a significant impact on the environmental performance of non-SOEs but has no significant impact on SOEs. Our findings further provide empirical evidence for the study of ownership structure on enterprise environmental performance (57). Compared with non-SOEs, SOEs are more politically connected (30). For example, Chinese SOEs have easier access to bank credit facilities and government subsidies. However, while enjoying the benefits, state-owned enterprises also need to make concessions and shoulder more social responsibilities (31). Therefore, the environmental performance pressure of SOEs mainly comes from the government, and due to the resource advantages of SOEs, external stakeholders are less able to exert pressure on them, according to resource dependence theory. Accordingly, EPLI has no apparent effect on the environmental performance of SOE. While the environmental performance pressure of non-SOEs comes from different stakeholders, the stakeholder pressure brought by EPLI will significantly improve the corporate environmental performance.

## Conclusion

### Theoretical Contributions

Scholars have paid much attention to the research on corporate environmental performance in recent years (12). The influence of factors such as corporate governance structure, external pressure and policy tools on environmental performance has been discussed in the existing literature (9, 12, 13). However, as an innovative financial product related to environmental protection, EPLI's impact on corporate environmental performance has received little attention, especially with little literature providing evidence from an empirical perspective. We systematically analyze the relationship changes between corporates and external stakeholders after the purchase of EPLI and further analyze the impact of relationship changes on enterprise environmental performance. In addition, we found the moderating effect of public visibility and ownership structure. Therefore, our findings provide a new perspective to studying the mechanisms that influence corporate environmental performance.

First, this study promotes the research on environmental performance and expands the application connotation of stakeholder theory by identifying the impact of EPLI on environmental performance and its mechanism. Previous studies have examined the impact of measures taken by companies such as ISO 14001 certification and environmental management systems (EMS) on environmental performance (13, 58). However, scholars' research on EPLI mainly focuses on its insurance attribute, and most studies on the environmental effects of EPLI are currently focused on qualitative case studies (16, 25). The influence of EPLI on environmental performance from the perspective of the institutional attribute is worth exploring. Our research attempted to explain the relationship between EPLI and corporate environmental performance with reference to stakeholder theory. Specifically, we first identify the stakeholder relationship between corporates and other external entities in the EPLI system and then further analyze the role of the stakeholder relationship in improving corporates' environmental performance. Based on stakeholder theory, the existing literature often analyzed the environmental pressure of stakeholders from the perspective of stakeholder salience (59), while the analysis of stakeholder salience in the EPLI system is theoretically lacking. This study discussed the stakeholder salience of two key stakeholders, the government and insurance companies, and confirmed their positive effect on environmental performance through empirical methods. Furthermore, this study also combined with the stakeholder-agency theory to explore how EPLI can increase the environmental pressure of stakeholders on the enterprise and further expand the connotation of EPLI institutional effect. Overall, this study fills in the research gap of factors influencing environmental performance from the perspective of EPLI and expands the application scope of stakeholder theory.

Second, the influence of public visibility on the environmental performance of corporates is considered in this study, which enriches the research on public visibility. As an important concept in stakeholder theory, previous literature has examined the effect of public visibility on corporate social responsibility (56, 60). However, no studies have focused on the factors that may affect the environmental effects of EPLI. From the perspective of agency theory, the opportunism behavior of the management will weaken the actual influence of stakeholder pressure on the enterprise. Social stakeholders will maintain a strong interest in companies with higher visibility, thus inhibiting managers' opportunistic tendencies. In this situation, the environmental pressure exerted by the EPLI system on the enterprise will be better translated into a higher level of environmental performance. Our findings revealed the significant positive impact of EPLI on environmental performance in companies with higher public visibility, providing a new insight for the study of public visibility.

Third, we contribute to the resource dependence theory by dividing the sample into state-owned and non-state-owned enterprise groups and examining the effect of EPLI on their environmental performance separately. Compared with the situation in other countries, the differences in political connections between China's SOEs and non-SOEs are greater (30). We analyzed the heterogeneity of environmental performance between SOEs and non-SOEs, and the results showed that EPLI was only effective in promoting corporate environmental performance in non-SOEs with weak political constraints. Therefore, the results of this study can provide evidence for corporate environmental performance under different resource constraints.

### Managerial Implications

Under the background that enterprises pay more and more attention to environmental social responsibility (61), the conclusions obtained in this paper can effectively and practically guide decision-makers to take green measures. This study revealed that EPLI could not only transfer the risk of environmentally responsible accidents (17), but also have a positive impact on a company's daily environmental performance. Specifically, the practical significance of this paper includes the following points.

First, the company can actively purchase EPLI for senior executives to encourage them to improve the environmental performance. EPLI is an effective way to motivate companies to improve their environmental performance for companies in heavily polluting industries. The company's decision-makers should realize that it is necessary to introduce external environmental pressure to improve the environmental performance of the company in the context of China's inadequate environmental laws and regulatory systems, and EPLI has the effect of increasing environmental pressure on company. In particular, under the modern corporate governance structure with the separation of ownership and management, EPLI introduces a multi-subject system, which, to a certain extent, intensifies the environmental pressure of stakeholder groups on the corporate, thus playing a role in regulating corporate behavior.

Second, the government can take administrative measures to force SOEs to implement EPLI, so as to enhance SOEs to actively fulfill their environmental responsibilities. Although the environmental effect of EPLI is satisfactory, the low insurance rate of enterprises is still a serious problem due to the imperfect environmental laws and regulations in China (25). Therefore, government enforcement measures can be adopted at the present stage for enterprises with serious environmental pollution. Because SOEs are facing more political pressure than non-SOEs, more attention can be paid to non-SOEs in the case of enforcement.

Third, environmental policy makers can adopt the strategy of forcing enterprises to disclose EPLI information to improve the environmental performance of enterprises. At present, the company's purchase of EPLI is not included in the scope of compulsory information disclosure for listed companies in China. However, the compulsory disclosure of this information may help EPLI to play its role in easing financing constraints. Furthermore, perfecting the information communication channels between companies and stakeholders is conducive to improving the companies' public visibility, which can promote a more significant improvement in environmental performance.

### Limitations and Future Directions

This study explains the relationship between EPLI and environmental performance from both theoretical and empirical perspectives. However, our study still has several limitations that need to be discussed. First, we used data from listed companies in heavily polluting industries for empirical testing, so the applicability of our results is limited to specific countries and industries. We have tried to obtain the company's insurance information from the public information disclosure (such as a financial report or social responsibility report). However, since the EPLI is not a compulsory disclosure of the listed company, the samples obtained by this method are generally inefficient. We believe that with the improvement of the information disclosure system of listed companies, follow-up research can be carried out based on larger sample size.

Second, our study only focused on the impact of whether a company has EPLI on environmental performance. For future research, more potential factors such as CEO characteristics (10, 62) need to be explored to influence the relationship between EPLI and environmental performance. In conclusion, it is hoped that this study can provide ideas for other studies and further discuss the microeconomic effects of EPLI. Future research can explore how EPLI and other measures to promote environmental performance, such as environmental regulation, can work together.

## Data Availability Statement

The datasets presented in this study can be found in online repositories. The names of the repository/repositories and accession number(s) can be found at: https://www.gtarsc.com/.

## Ethics Statement

Ethics approval for this research was not required as per institutional and national guidelines. Consent from all research participants was obtained by virtue of survey completion.

## Author Contributions

All authors listed have made a substantial, direct, and intellectual contribution to the work and approved it for publication.

## Funding

This paper was supported by the National Social Science Foundation of China (Grant No. 21BGL061), a major social science project of Tianjin Education Commission (No. 2019JWZD33), and Tianjin philosophy and social science planning project (TJGL20-026).

## Conflict of Interest

The authors declare that the research was conducted in the absence of any commercial or financial relationships that could be construed as a potential conflict of interest.

## Publisher's Note

All claims expressed in this article are solely those of the authors and do not necessarily represent those of their affiliated organizations, or those of the publisher, the editors and the reviewers. Any product that may be evaluated in this article, or claim that may be made by its manufacturer, is not guaranteed or endorsed by the publisher.
